# Treatment of medial-sided injuries in patients with early bicruciate ligament reconstruction for knee dislocation

**DOI:** 10.1007/s00167-020-06207-x

**Published:** 2020-08-30

**Authors:** Mikko A. Jokela, Tatu J. Mäkinen, Mika P. Koivikko, Joonas M. Lindahl, Jyrki Halinen, Jan Lindahl

**Affiliations:** 1 Department of Orthopaedics and Traumatology, Töölö Hospital, Helsinki University Hospital, Helsinki, Finland; 2 Department of Radiology, Helsinki Medical Imaging Center, Töölö Hospital, Helsinki University Hospital, Helsinki, Finland; 3 University of Helsinki, Helsinki, Finland; 4 Terveystalo Hospital, Helsinki, Finland

**Keywords:** Knee multiligament injury, Knee dislocation, KDIIIM, Knee posteromedial corner injury, Bicruciate ligament injury, Medial side injury

## Abstract

**Purpose:**

In knee dislocation with bicruciate ligament and medial side injury (KDIIIM), treatment method of medial side injuries is controversial. The purpose of this study was to evaluate the outcomes of non-operative treatment of proximal and midsubstance and operative treatment of distal avulsion medial collateral ligament (MCL) ruptures in patients with early bicruciate reconstruction.

**Methods:**

One-hundred and forty-seven patients with a knee dislocation and bicruciate ligament injury (KDII-KDV) were identified. Sixty-two patients had KDIIIM injury. Of these, 24 patients were excluded and 13 were lost to follow-up. With a minimum of 2 years of follow-up, IKDC2000 (subjective and objective), Lysholm and Tegner scores and stress radiographs were recorded.

**Results:**

Twenty-five patients were available for follow-up: 18 had a proximal or midsubstance grade-III MCL rupture (proximal MCL group) and 7 had a distal MCL avulsion (distal MCL group). In the proximal MCL and distal MCL groups, respectively, median IKDC2000 subjective scores were 80 (range 57–99) and 62 (range 39–87), and median Lysholm scores were 88 (range 57–99) and 75 (range 40–100). The median medial opening (side-to-side difference) was 2.4 mm (range 0.1–9.2) in the proximal MCL group and 2.5 mm (range 0.2–4.8) in the distal MCL group.

**Conclusion:**

We found acceptable recorded outcomes in patients who underwent non-operative treatment of proximal and midsubstance grade-III MCL rupture and operative treatment of distal MCL avulsion with early bicruciate ligament reconstruction.

**Level of evidence:**

Level IV

## Introduction

Knee dislocation is a rare injury typically caused by high-energy trauma, but it can occur with low-energy insult during sports or even in a same-level fall [37]. Knee dislocation leads most often to the complete rupture of both the anterior and posterior cruciate ligaments (ACL and PCL, respectively), and rupture of both cruciates thus could be considered a type of knee dislocation [36]. Concomitant medial side ligamentous injuries are often present in knee dislocations [20]. The main structures medial to the knee joint are the proximal and distal divisions of the superficial medial collateral ligament (sMCL), the meniscofemoral and meniscotibial divisions of the deep medial collateral ligament (dMCL), and the posterior oblique ligament (POL) [11, 39]. A medial side injury combined with bicruciate ligament injury (KDIIIM according to Schenck’s classification) consists of MCL and POL injuries [38].

Solitary grade-III MCL injury can be treated non-operatively with good knee function and stability [16, 28]. Evidence also suggests that MCLs do not need to be repaired in combined ruptures of the MCL and ACL if the ACL is repaired at an early stage [12, 13, 39]. Operative treatment of both cruciate ligaments in the acute phase also seems to lead to good outcomes [7, 9, 14, 29]. Consensus is lacking, however, on treatment of KDIIIM injuries. Good results have been reported with both non-operative and operative treatments of medial side injuries and bicruciate ligament reconstruction in the acute phase [3, 7, 22, 34, 38]. There is a risk of residual valgus laxity with conservative treatment of distal MCL avulsion injury [42] or in the presence of an MCL Stener-like lesion [5, 40], and operative treatment is recommended for a grade-III distal tear or tibial avulsion of the MCL [2, 25, 26]. Repair of acute or chronic MCL rupture seems to carry a higher risk for poor outcome compared with reconstruction [19, 32]. Repair of a proximal medial side injury might carry a greater risk for post-operative knee stiffness than non-operative management [30], and knee stiffness is a risk after MCL repair with simultaneous cruciate reconstruction [27]. However, literature is scarce regarding treatment of acute knee dislocation with bicruciate and medial side injury [20, 24, 35, 38].

Our hypothesis was that in knee KDIIIM injuries, proximal or midsubstance grade-III MCL and POL injuries can be treated non-operatively when bicruciate reconstruction is performed in the acute phase, with outcomes comparable to those in earlier published results of operatively treated MCL injuries. An exception would be distal sMCL avulsion injury (Stener lesion), which should be treated operatively. To test this hypothesis, we conducted a retrospective follow-up study of KDIIIM patients to evaluate clinical and radiological outcomes. To our knowledge, Telos stress radiographs have not been used previously in this setting to evaluate the outcome. The aim of this study was to report results of conservative treatment of proximal and midsusbtance MCL injury and surgical treatment of distal MCL injury, not to make direct comparison of these two.

## Materials and methods

Ethical approval for this study was obtained from the Ethics committee of Helsinki University Hospital (registration number 105/13/03/02/2015).

Between 2004 and 2014, a total of 147 patients with knee dislocation and bicruciate ligament injury (KDII-KDV) were treated at Töölö Hospital, Helsinki University Hospital. Töölö hospital is a level 1 trauma center and tertiary referral center for patients with severe injuries, including knee dislocations, with a catchment area of 1.8 million people.

**Fig. 1:**
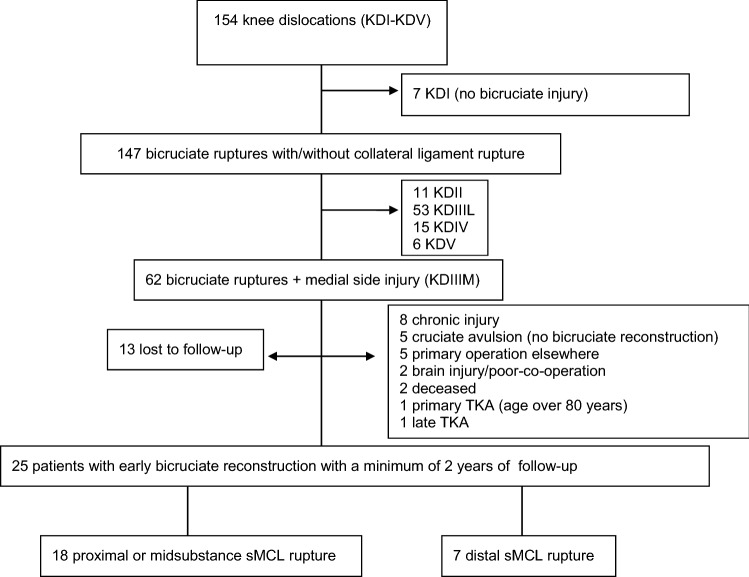
Flowchart describing the selection process for 154 knee dislocations (KDI-KDV) treated from 2004 to 2014. TKA, total knee arthroplasty

An inclusion criterion was an acute KDIIIM injury according to Schenck’s classification, treated with arthroscopic bicruciate ligament reconstruction and non-operative or operative treatment of medial knee structures in a skeletally mature patient. Exclusion criteria were bilateral knee ligament injury, combined medial and lateral side injury (KDIV) or fracture dislocation (KDV), operative treatment done primarily at another institution, any previous surgery on the affected knee, and severe head injury or poor co-operation. Figure 1 gives an overview of the inclusion/exclusion process. All patients gave signed informed consent.

Sixty-two patients had a KDIIIM injury, of whom 24 were excluded (Fig. 1) and 13 were lost to follow-up. Of the remaining 25 patients, 5 were injured in a high-energy trauma (3 pedestrians hit by a car, 2 motorcycle accidents) and 20 in a low-energy trauma (13 sports related, 5 same-level fall, 2 fall < 2 m). Seven patients presented with a knee dislocation in the emergency room and were reduced. The remaining patients had a knee dislocation reduced either spontaneously or at the scene of trauma. Primary diagnosis was made by clinical examination. The Lachman test and anterior and posterior drawer tests were positive, and the medial joint space opening to valgus stress testing in extension (5 and at 20) was more than 10 mm. Patient characteristics are presented in Table 1.

**Table 1:** KDIIIM patient characteristics (*n* = 25)

	Proximal and midsubstance MCL (*n* = 18)	Distal MCL (*n* = 7)
Sex F/M	5F, 13 M	3F, 4 M
Age, years, median (range)	39 (21–64)	49 (17–67)
Follow-up time, months; median (range)	98 (40–145)	66 (24–82)
Surgical timing, days; median (range)	17 (6–28)	11 (5–38)
Trauma energy, low/high	14 low, 4 high	5 low, 2 high
BMI, median (range)	29 (19–39)	29 (24–37)

Knee magnetic resonance imaging (MRI) was performed on every patient on an average 3 days ( range, 1–11 days) from assessment at our institution. The delay on knee-MRI on some patients was due to other more severe trauma. Angiographic studies (CT angiography, conventional angiography) were done selectively based on the clinical suspicion of vascular injury. In this study, three patients underwent CT angiography and one patient conventional angiography.

On clinical evaluation, all patients had complete ACL, PCL, and medial side injuries. No vascular or nerve injuries were detected in this KDIIIM series. Seven patients had a minor impression on either the femoral or tibial articular surface. Eight patients had meniscal injury (3 medial, 3 lateral, 2 both). Table 2 provides information on associated injuries.

**Table 2:** Associated injuries in KDIIIM injuries (*n* = 25)

	Proximal and midsubstance MCL (*n* = 18)	Distal MCL (*n* = 7)
Meniscal injury		
Medial	2	1
Lateral	1	2
Both	2	0
Chondral lesion		
Femoral	1	2
Tibial	3	3

All patients were treated with hinged knee brace until the definite treatment, except in three patients who had provisional stabilization of the knee joint carried out with a spanning external fixator (9–17 days).

Five surgeons were involved in this study. All of these were senior orthopaedic and trauma surgeons with special training of knee multiligament injuries.

The patients were treated with bicruciate reconstruction on an average 19 days from injury (range, 5–38 days). The anatomic location of the MCL injury determined whether the treatment was non-operative (proximal or midsubstance injury) or surgical (distal injury). In 18 patients, medial side injuries (proximal and midsubstance sMCL) were treated non-operatively (the proximal MCL group). In seven patients, medial side injury (distal sMCL) was treated operatively (the distal MCL group). The ACL was reconstructed with transtibial drilling of the femoral tunnel in 12 patients and with anteromedial portal in 13 patients (7 autograft, 18 allograft). All PCL reconstructions were performed with transtibial technique (11 single-bundle and 14 double-bundle, 6 auto- and 19 allografts). Meniscal injuries were treated with partial meniscectomy (5 in the proximal MCL group; 1 in the distal MCL group) or with meniscal repair (2 in the distal MCL group).

Once the treatment strategy was decided, no valgus stress was applied to the knee, to avoid any further damage to the MCL.

Final clinical stability assessment of knee ligaments was performed under anaesthesia in the operating room to verify MRI findings. Again, no valgus stress was applied to the knee with proximal or midsubstance MCL injury seen in MRI to avoid any further damage to the healing MCL. ACL and PCL were reconstructed arthroscopically with tendon autografts or allografts. After bicruciate ligament reconstruction, the clinical valgus laxity in full extension was normal in the proximal MCL group. In distal sMCL injuries, repair (*n* = 6) or reconstruction using modified Bosworth technique with tendon allografting (*n* = 1) was performed.

All patients were treated with a hinged knee brace for a minimum of 12 weeks post-surgery. Full range of motion (ROM) was allowed after 2–4 weeks depending on the patient-related factors: ROM was initiated typically at 2 weeks in non-obese patients and at 4 weeks in obese patients as the knee brace might provide inadequate initial support. In cases where concomitant meniscal injuries were repaired (two patients in the distal MCL group), ROM was 0–60° for the first 4 weeks and 0–90° for the following 2 weeks. In weeks 0–4, partial weight bearing (15 to 20 kg) was allowed, and for weeks 5–6 half weight bearing was allowed. After 6 weeks, full ROM and weight bearing were allowed, and after 12–16 weeks, the brace was discontinued. Muscle rehabilitation was initiated with closed kinetic chain exercises. Return to sport activities was allowed 12 months after the operation.

**Fig. 2:**
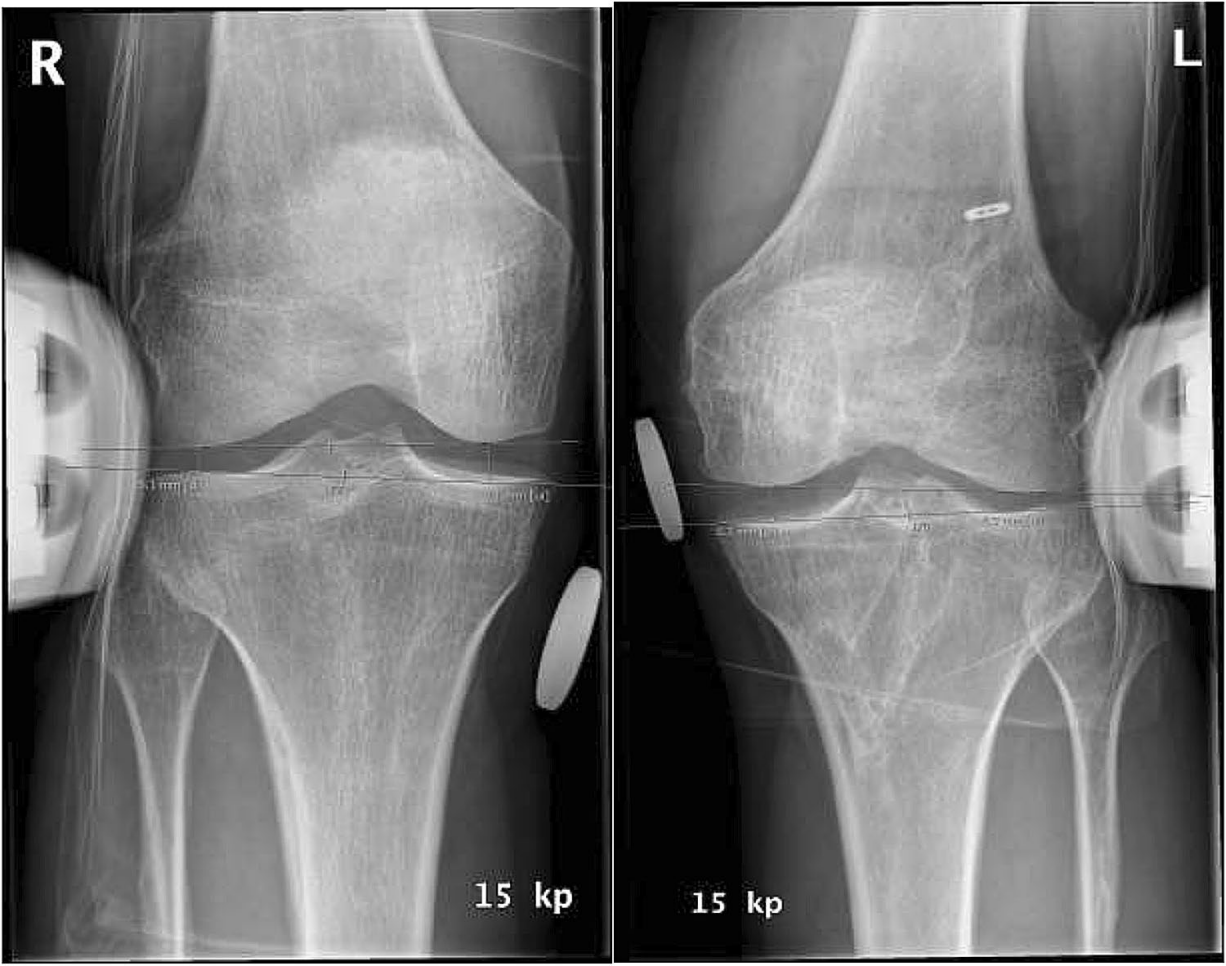
Valgus stress radiographs taken with knee in 20**°** flexion of uninjured and injured knees 3 years after bicruciate reconstruction (left knee) with Telos device on a patient with proximal grade-III MCL rupture. Telos radiographs show a 2.2 mm side-to-side difference, representing a good radiological outcome

One independent examiner (M.J.) performed all of the assessments during the final follow-up. Lysholm and Tegner scores, as well as International Knee Documentation Committee (IKDC2000) subjective and examination forms, were recorded. Clinical evaluation was performed according to the IKDC2000 objective examination form [1, 15]. Anterior-posterior laxity was measured radiographically with a Telos device (Telos machine, SAMO, Bologna, Italy), according to guidelines [10, 33]. Telos device valgus–varus stress radiographs were also obtained accordingly, and the side-to-side difference was calculated by comparison with the contralateral uninjured knee [17] (Fig. 2). Varus and valgus laxity stress tests were performed with the knee flexed 20°. All radiographs were evaluated by an experienced musculoskeletal radiologist (M.K.) with a measurement accuracy of one decimal. The IKDC valgus subscore was used for objective MCL stability assessment; IKDC grade A indicates a medial joint opening of 0 to 2 mm, grade B of 3 to 5 mm, grade C of 6 to 10 mm, and grade D of > 10 mm. Knee ROM was measured with a goniometer. Knee stiffness (arthrofibrosis) was defined as a knee extension deficiency of more than 10 degrees and flexion deficiency more than 20 degrees.

### Statistical analysis

In the current study, the aim was not to make any statistical comparison between the results of conservative treatment of proximal and midsubstance MCL injury and surgical treatment of distal MCL injury and power analysis is not applicable. For applicable analysis SPSS 24 (IBM Corp. IBM SPSS Statistics for Macintosh, Version 24.0. Armonk, NY: IBM Corp.) was used.

## Results

Twenty-five patients were available for final follow-up: 18 in the proximal MCL group and 7 in the distal MCL group.

### Patient-related outcomes

The median IKDC2000 subjective score was 80 (range 57–99) in the proximal MCL group and 62 (range 39–87) in the distal MCL group. The median Lysholm score was 88 (range 57–99) in the proximal MCL group and 75 (range 40–100) in the distal MCL group. Subjective and clinical results are presented in Table 3.

**Table 3:** Subjective results of KDIIIM injuries (*N* = 25)

	Proximal and midsubstance MCL (*n* = 18)	Distal MCL (*n* = 7)
IKDC2000 subjective, median (range)	80 (57–99)	62 (39–87)
Lysholm score, median (range)	88 (57–99)	75 (40–100)
Tegner activity level, median (range)	3 (2–7)	3 (1–6)

### Stress radiographs and objective results

The median medial knee laxity in the valgus stress test measured from Telos radiographs (side-to-side difference) was 2.4 mm (range 0.1–9.2) in the proximal MCL group and 2.5 mm (range 0.2–4.8) in the distal MCL group.

Fifteen patients in the proximal/midsubstance group had a medial gapping side-to-side difference < 5 mm, corresponding to normal or nearly normal medial stability (A or B, respectively) according to IKDC2000 criteria (Table 4).

**Table 4:** Stress radiographs and objective IKDC2000 results of KDIIIM injuries

Stress radiograph side-to-side difference	Proximal and midsubstance MCL (*n* = 17*)	Distal MCL (*n* = 6*)
Medial, mm median (range)	2.4 (0.1–9.2)	2.5 (0.2–4.8)
0–2 mm (normal)	6	2
3–5 mm (nearly normal)	9	4
6–10 mm (abnormal)	2	0
> 10 mm (severely abnormal)	0	0
Antero-posterior, mm median (range)	2.4 (0.3–11.2)	5.6 (0.9–15.5)
0–2 mm (normal)	8	1
3–5 mm (nearly normal)	6	4
6–10 mm (abnormal)	2	1
> 10 mm (severely abnormal)	1	0
IKDC2000 objective	(*n* = 18)	(*n* = 7)
A (normal)	4	0
B (nearly normal)	6	3
C (abnormal)	8	2
D (severely abnormal)	0	2

*One patient in both groups were excluded from radiological analysis

### Return to sports

**Fig. 3:**
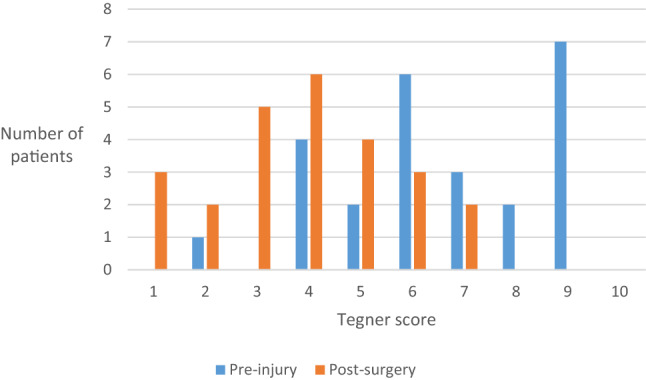
Patient Tegner scores presented pre-injury and post-surgery (n=25)

The highest pre-injury Tegner level was 9/10 (seven patients). There were no professional athletes among the patients. Four patients with proximal MCL injury did return at a pre-injury Tegner score level, while 21 patients returned at a lower level. No patient was on sick leave or disability pension due to knee problems at the time of clinical assessment (Tegner 0). The patient-reported changes in Tegner scores are presented in Fig. 3.

### Complications

There were three re-operations: one in the proximal MCL group and two in the distal MCL group. In the proximal MCL group, medial side laxity at 1 year after the primary operation was managed with MCL reconstruction with a tendon allograft. The distal MCL group re-operations included one arthroscopic lavage because of acute (8 days post-operative) infection and one MCL re-fixation. The patients with medial side re-operation were excluded from radiological analyses.

No patient had an extension deficit. Two patients had a flexion deficit > 20 degrees, and both of them had a distal MCL tear (distal MCL group).

## Discussion

The most important finding of the present study was that acceptable subjective, clinical, and radiological outcomes can be achieved with non-operative treatment on proximal or midsubstance MCL injury when concomitant bicruciate injury is treated with reconstruction in the acute phase in KDIIIM injuries. Based on the valgus stress test, most knees were classified as stable. Medial stability according to the IKDC2000 score showed 89% normal or nearly normal (grade A or B) knees. One patient had an MCL reconstruction 1 year later for persistent instability. This study provides a schematic approach for treatment of medial-sided injuries in knee dislocations.

Operative treatment is widely recommended for distal MCL avulsion injury in the presence of an MCL Stener-like lesion, and when the MCL is trapped in the joint [25, 40, 42]. The risk of residual valgus laxity after conservative treatment is high. All these distal MCL injuries were treated operatively. At follow-up, medial stability according to the valgus stress test and IKDC2000 scores was normal or nearly normal (grade A or B) in six of seven patients. One patient had an MCL refixation later on. Median Lysholm scores, Tegner activity levels, and IKDC2000 subjective scores were slightly lower than in the proximal MCL group, but acceptable.

Previous studies have indicated that a grade-III MCL rupture can be treated conservatively when a concomitant ACL rupture is reconstructed [8, 12, 13, 24, 41]. If valgus laxity remains after initial treatment with a hinged brace (representing a chronic injury), the rupture is most often in the distal MCL [26, 42]. In chronic medial side injury, reconstruction is recommended [23, 39] because late repair of the medial side might lead to an inferior outcome [19]. Combined MCL and PCL injury is rare [3, 18, 31], with no clear consensus on treatment strategies [4, 38].

In a recent study by Barrett et al. [3], the outcomes of MCL reconstructions with tendon allografts were published. There were 32 patients with MCL injury and 12 had KDIIIM injury. For all types of knee dislocations, the average IKDC score was 67.6. Knee dislocation grade inversely correlated with clinical outcome measures post-surgery, and in the KDIIIM group the average IKDC score was only 56.6.

Few studies have addressed outcomes of non-operative treatment of acute medial side rupture with bicruciate injury [20, 38]. Fanelli et al. [7] reported results of this subset of injury, finding that seven out of eight patients with bicruciate and medial side injury treated with bicruciate reconstruction and bracing were stable in knee valgus stress, with a mean Lysholm score of 91.2. However, these authors obtained no valgus stress radiographs, and specific results of non-operatively treated MCL injuries in their patient subset were not reported.

In a more recent study, Werner et al. [38] evaluated medial side injury in knee dislocations in 65 patients, 16 with a conservatively treated medial side injury (2 KDIIIM injury, 14 KDIV injuries) and 49 patients with medial side injuries either repaired or reconstructed. Of the total, 35 patients were available for final follow-up. The overall Lysholm score in KDIIIM patients was 88, and these authors reported no results for patients treated non-operatively for medial side injury.

Two recent studies compared the outcomes of operative treatment of medial and lateral side injuries in knee multiligament injury. Tardy et al. [34] reported results for 19 patients with posteromedial corner repair or reconstruction. All patients were operated on in the acute phase. The subjective IKDC score was 81, and the Lysholm score was 89. However, only 13 of the patients had a bicruciate knee injury. No radiological examinations were reported. In a study by King et al. [19], operative treatments of medial and lateral side injuries were compared. Twenty-four patients with KDIIIM injury were available at last follow-up. The reported mean subjective IKDC was 62.1, and the mean Lysholm score was 64.7. The low scores might be attributable to the fact that these were chronic injuries, with a mean time of 9.8 months from injury to surgery.

The scores applied in this study (Lysholm score, Tegner activity scale, and IKDC2000 scores) are widely used in evaluating knee function. The knee stability was assessed with stress radiographs to improve the quantification of medial gapping [21]. The non-operative treatment of acute medial side rupture with a hinged brace was found to provide acceptable clinical and radiological results. In the non-operative group (proximal and midsubstance MCL rupture; proximal MCL group), the overall results were similar to those for patients treated with medial side reconstruction in previous studies [23, 34, 38]. In this group, the stress radiographs showed a medial opening side-to-side difference of < 5 mm, corresponding to an IKDC2000 result of A or B (normal or nearly normal) in 16 of 18 patients. The average Lysholm score of 88 (good) and IKDC2000 subjective score of 80/100 are strong indicators of patient satisfaction. Risk for arthrofibrosis is higher with medial side repair [6, 32], and our results support this finding: flexion deficit was detected only in the operatively treated distal MCL group (2 patients, 29%). The overall rate for stiffness was similar compared with previous reports [32, 38], although no additional surgery for stiffness was needed in our study.

Knee dislocation is a rare injury, and acquiring enough patients for a proper study requires a relatively long period of time. In 11 years, we identified 62 patients with KDIIIM injury, and 24 of these patients were excluded. Because only 25 patients out of 38 were available for final follow-up, there is a risk of selection bias. Since one group of patients was treated non-operatively (proximal and midsubstance MCL injury) and other operatively (distal MCL injury), direct comparison between the groups is not possible. The mean follow-up time was 105 months, so conclusions about the development of posttraumatic osteoarthrosis are difficult to draw.

The main strength of this study is the relatively large study group with homogeneous injury, achieved by including only patients with acute medial side injury and bicruciate reconstruction and excluding patients with lateral side (KDIV) and chronic injuries, concomitant fractures (KDV), and any previous surgery on the affected knee. All patients were treated following the same treatment protocol. Objective evaluation of medial side gapping was achieved with Telos device stress radiographs. To our knowledge, this study is the largest to date of acute KDIIIM patients with reported results of valgus stress radiographs.

## Conclusions

Treatment of acute knee dislocations with bicruciate and complete medial side injuries remains controversial. These findings suggest that acceptable functional and radiological results can be achieved with non-operative treatment of proximal and midsubstance grade-III medial collateral ligament rupture with a hinged brace when bicruciate reconstruction is performed in the acute phase. In addition, acceptable outcomes were seen after operative treatment of distal avulsion medial collateral ligament rupture with early bicruciate ligament reconstruction.
